# Metformin-induced energy deficiency leads to the inhibition of lipogenesis in prostate cancer cells

**DOI:** 10.18632/oncotarget.3404

**Published:** 2015-03-10

**Authors:** Camille Loubière, Thomas Goiran, Kathiane Laurent, Zied Djabari, Jean-François Tanti, Frédéric Bost

**Affiliations:** ^1^ INSERM, C3M, U1065, Team Cellular and Molecular Physiopathology of Diabetes and Obesity, Nice 06200, France; ^2^ University of Nice Sophia Antipolis, C3M, U1065, Nice 06200, France

**Keywords:** prostate cancer, lipogenesis, metformin, cancer metabolism, ATP

## Abstract

The deregulation of lipid metabolism is a hallmark of tumor cells, and elevated lipogenesis has been reported in prostate cancer. Metformin, a drug commonly prescribed for type II diabetes, displays antitumor properties. Here, we show that metformin inhibits lipogenesis in several prostate cancer cell lines. In LNCaP cells, this effect parallels the decrease of key lipogenic proteins: ACC (acetyl-CoA carboxylase), FASN (fatty acid synthase) and SREBP1c (sterol regulatory element binding protein-1c), whereas there is no modification in DU145 and PC3 cells. Despite the relatively high level of lipogenic proteins induced by the overexpression of a constitutively active form of SREBP1c or treatment with androgens, metformin is still able to inhibit lipogenesis. Metformin does not alter the concentration of malonyl-CoA (the fatty acid precursor), and it only slightly decreases the NADPH levels, which is a co-factor required for lipogenesis, in LNCaP. Finally, we show that the inhibitory effect of metformin on lipogenesis is primarily due to a cellular energy deficit. Metformin decreases ATP in a dose-dependent manner, and this diminution is significantly correlated with the inhibition of lipogenesis in LNCaP and DU145. Indeed, the effect of metformin is linked to changes in the ATP content rather than the regulation of protein expression. Our results describe a new mechanism of action for metformin on prostate cancer metabolism.

## INTRODUCTION

Prostate cancer is the second leading cause of death by cancer in men. Most prostate cancer-related deaths are due to advanced stages of the disease and the formation of metastases. Patients with metastatic prostate cancer initially respond to anti-androgen therapy for a median time of 12 to 18 months [1] and then become resistant to the treatment. Unfortunately, chemotherapeutic treatments display modest results, with a median survival of 18 months in patients treated with docetaxel [2]; therefore, there is an urgent need for additional therapy. In the era of targeted therapy, there is a growing interest in drugs targeting metabolic pathways. The tumor growth phase is characterized by a high metabolic demand. Prostate tumors activate glycolysis in the advanced stages, whereas an increase in lipogenesis is observed during the early and late stages of the disease. The deregulation of lipid metabolism, or more specifically, the increased rate of lipogenesis, is essential to maintain the tumor cells growth rate, and inhibitors of lipid synthesis have been shown to inhibit cancer cell proliferation [3]. Fatty acid synthase (FASN) and acetyl-CoA carboxylase (ACC) are two key enzymes that regulate lipogenesis. ACC catalyzes the formation of acetyl-CoA into malonyl-CoA, the precursor of newly synthesized fatty acids, and its activity is negatively regulated by its phosphorylation by AMP-activated protein kinase (AMPK). Sterol Regulatory Element-Binding Protein 1c (SREBP1c) controls the expression of FASN, which catalyzes the synthesis of fatty acids from malonyl-CoA. Androgens and the Akt/mTOR pathway have been shown to stimulate lipogenesis through the direct control of the expression of SREBP1c, FASN and ACC.

Metformin is a widely used anti-diabetic drug prescribed to more than 120 million people worldwide [4, 5]. In agreement with retrospective epidemiological studies, diabetic patients treated with metformin display a reduction in their cancer incidence and cancer-related mortality [6–9]. Metformin inhibits cancer cell proliferation and decreases tumor growth in many animal models [10–13]. In addition, metformin sensitizes tumor cells to classical anti-cancer agents, such as disatinib [14]. Metformin alters cancer cell metabolism and it inhibits mitochondrial complex 1 and the mTOR pathway, which is a major regulator of cell metabolism and proliferation. However, the role of metformin on lipid metabolism in cancer cells is poorly understood.

Here, we show that metformin exerts a strong inhibitory effect on lipogenesis in prostate cancer cells. The overexpression of a constitutively active form of SREBP1c, as well as androgen treatment, increases the expression of FASN and ACC and strongly stimulates lipogenesis. Nevertheless, metformin hampers this effect. Finally, we demonstrate that metformin does not affect the malonyl-CoA or NADPH content but instead induces an energy deficiency that impairs lipogenesis.

## RESULTS

### Metformin inhibits lipogenesis in prostate cancer cells

**Figure 1:**
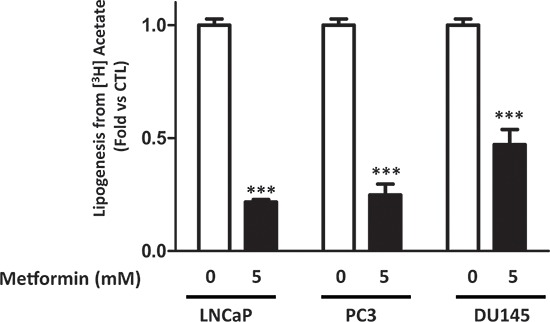
Metformin inhibits lipogenesis in prostate cancer cells The cells were treated for 24 h with 5 mM metformin before measuring the incorporation of the [^3^H] acetate into the lipids, as described in the Materials and Methods section. The results are representative of four independent experiments, ***p < 0.005.

We analyzed the effect of metformin on lipogenesis in three prostate cancer cell lines (LNCaP, DU145 and PC3). The cells were treated with 5 mM metformin, and the lipogenesis was quantified by measuring the incorporation of [^3^H] acetate into the fatty acids and triglycerides as described in the material and methods. Metformin inhibited the lipogenesis in the three cancer cell lines, with a stronger effect in the LNCaP cells (80% inhibition) after 24 h, and a reduction of 75 and 50% was observed in PC3 and DU145, respectively (Fig. 1). To confirm these observations, we incubated the cells with [^3^H] glucose, one of the precursors of fatty acids. Despite the fact that metformin increases glucose uptake in prostate cancer cells [17], we showed that it inhibits lipogenesis using the labeled glucose (Fig. S1). We also found that phenformin, a biguanide and analogue of metformin, strongly inhibits lipogenesis (Fig. S2). These results highlight the strong inhibitory effect of biguanides on the lipogenesis in prostate cancer cells.

**Figure 2:**
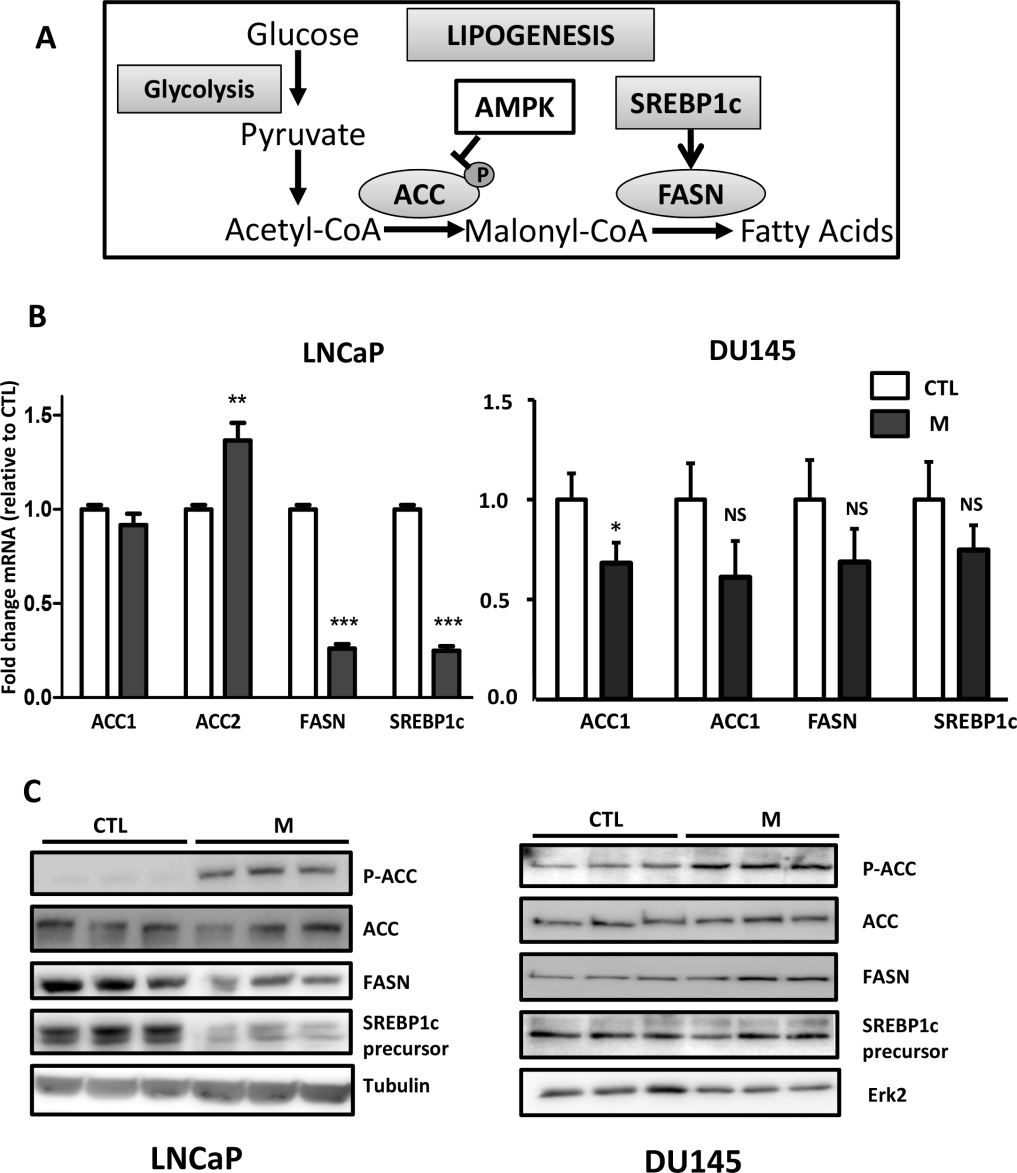
The effect of metformin on the lipogenic proteins **(A)** A simplified schematic representation of lipogenesis. ACC: acetyl-CoA carboxylase, FASN: fatty acid synthase, AMPK: AMP activated protein kinase. **(B)** The cells were treated with 5 mM metformin (M) for 24 h, and the mRNA levels of ACC, FASN and SREBP1c were determined as described in the Materials and Methods section. **p < 0.01; ***p < 0.005. **(C)** An immunoblot of the indicated proteins in the cells treated with 5 mM metformin for 24 h (M). The blots are representative of three independent experiments.

Metformin activates AMPK, which phosphorylates and inhibits ACC, and metformin downregulates mTORC1, which controls the expression of SREBP1c. We asked whether the effects of metformin on lipogenesis are related to a decrease in the expression of these lipogenic proteins. We analyzed the expression of SREBP1c, FASN and ACC (Fig. 2A). As expected, metformin induces the phosphorylation of ACC at Ser^79^, and it induces a strong decrease in the SREBP1c and FASN mRNA and proteins in the LNCaP cells, but it did not alter the expression of the ACC proteins after 24 h (Fig. 2B, 2C). We also analyzed the SREBP1c activity via transient transfections of a FASN promoter-luciferase reporter plasmid containing the SREBP1c responsive element. Consistent with the inhibitory effect of metformin, we found that the SREBP1c activity was drastically reduced in the LNCaP cells treated with metformin (Fig. S3).

Interestingly, although we still observed the phosphorylation of ACC at Ser^79^, we did not observe any significant change in the lipogenic protein expression in the DU145 and PC3 cells treated with metformin (Fig. 2B, 2C and data not shown). These results suggest that the anti-lipogenic effects of metformin in DU145 and PC3 are not due to the modification of the levels of the key proteins in lipogenesis.

### Constitutively active SREBP1c does not reverse the anti-lipogenic effects of metformin in LNCaP cells

**Figure 3:**
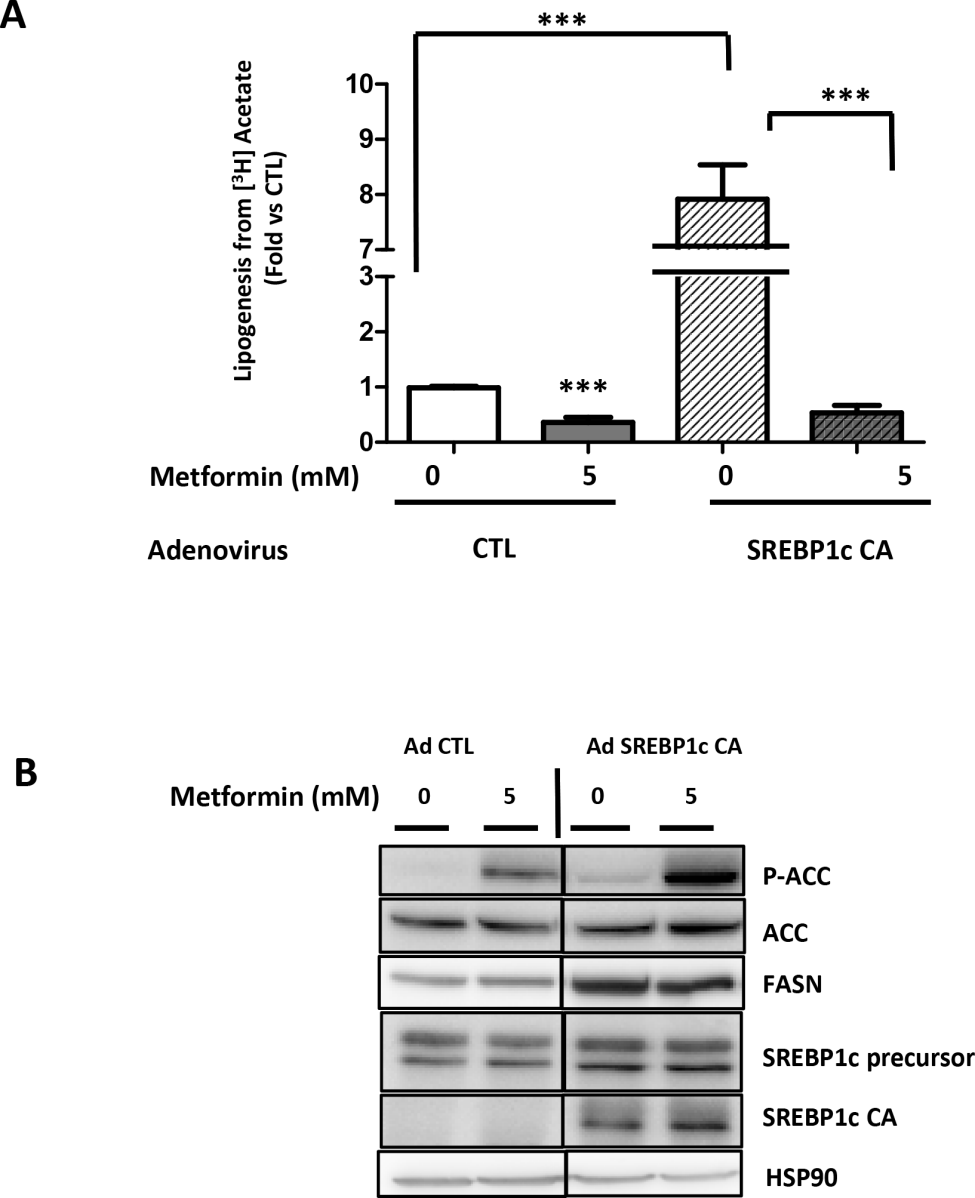
Metformin represses SREBP1c-stimulated lipogenesis **(A)** LNCaP cells were infected with a control adenoviral construct (AdCTL) or an adenoviral construct expressing the constitutive form of SREBP1c (AdSREBP1c CA). After 24 h, the cells were treated with 5 mM metformin (M) for 24 h, and the lipogenesis was observed using [^3^H] acetate as previously described. **(B)** An immunoblot of the key lipogenic proteins in the LNCaP cells treated as described in A. **p < 0.01; ***p < 0.005.

In order to clarify the role of the lipogenic proteins in the action of metformin, we expressed a constitutively activated form of SREBP1c (SREBP1c CA) in the LNCaP cells [18]. As expected, the SREBP1c expression strongly stimulates lipogenesis by 8-fold and the metformin inhibited this effect (Fig. 3A). The SREBP1c CA expression increased the FASN protein level, and the metformin did not affect this relatively high level of FASN (Fig. 3B). Our results demonstrate that despite a high level of FASN expression, the metformin was still able to hamper lipogenesis.

### Metformin inhibits androgen-induced lipogenesis

**Figure 4:**
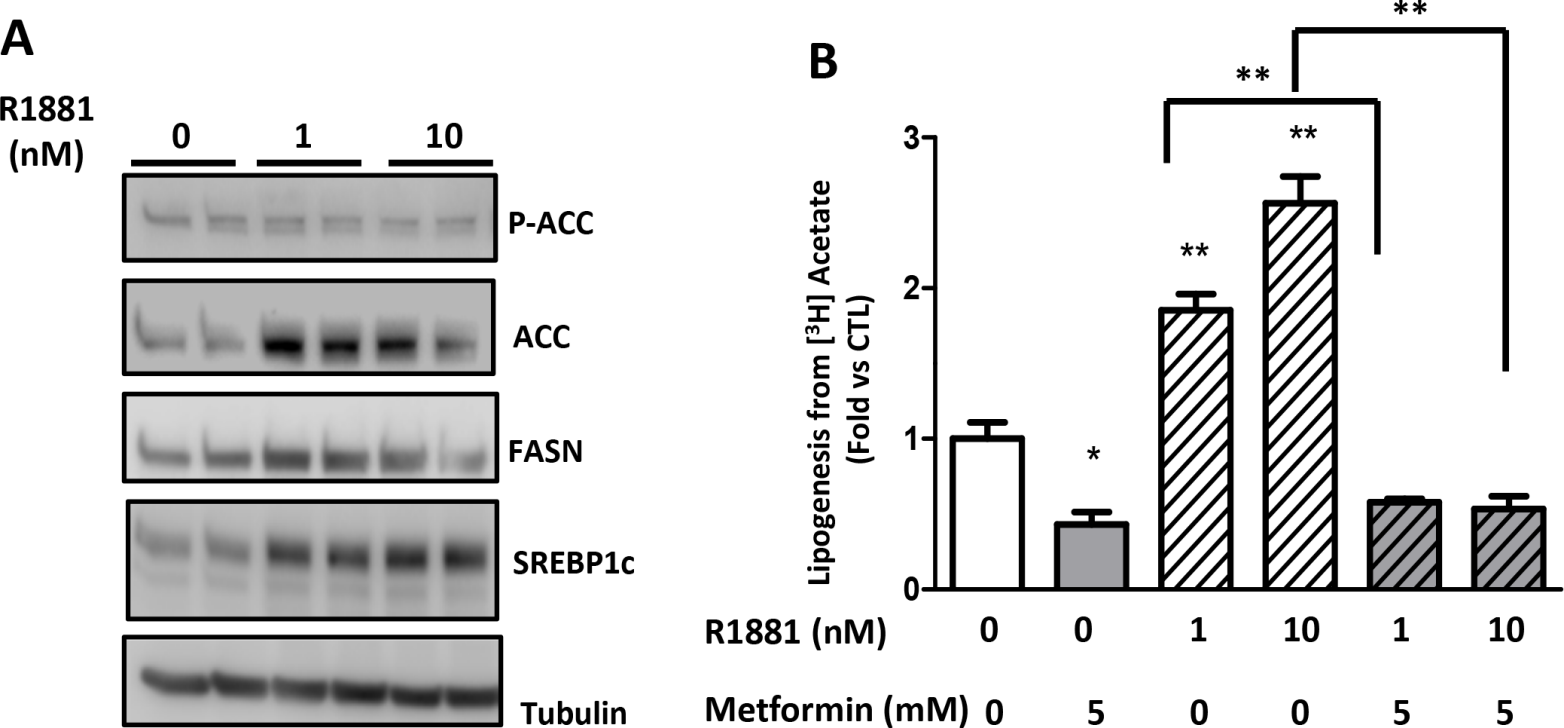
Metformin inhibits androgen-stimulated lipogenesis **(A)** An immunoblot of the lipogenic proteins in the LNCaP cells treated with 1 or 10 nM R1881 for 48 h and 5 mM metformin for 24 h. **(B)** The LNCaP cells were treated for 48 h with 1 or 10 nM R1881 and then with 5 mM metformin for 24 h before the quantification of the lipogenesis. **p < 0.01; ***p < 0.05.

Androgens control the growth and progression of prostate cancer; they stimulate lipogenesis and increase the expression of lipogenic enzymes in prostate cancer cells [19]. In a more physiological approach, we stimulated lipogenesis using R1881, a non-aromatizable synthetic androgen, and asked whether metformin affects the androgen-induced lipogenesis in the androgen-sensitive cell line LNCaP. The R1881 increased the ACC, FASN and SREBP1c protein expression after 48 h (Fig. 4A). Accordingly, the lipogenesis was upregulated after the addition of R1881 at 1 and 10 nM, but once again, the metformin inhibited the lipogenesis induced by the androgens (Fig. 4B). Altogether, our results demonstrate two different methods by which metformin inhibits lipogenesis independent of the level of lipogenic proteins.

### Metformin does not affect the malonyl-CoA concentration in prostate cancer cells

**Figure 5:**
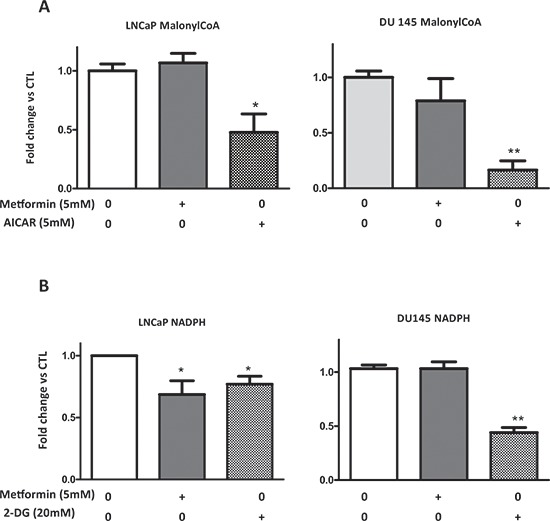
Metformin does not affect the malonyl-CoA and NADPH concentration **(A, B)** LNCaP and DU145 were treated with 5 mM metformin, 5 mM AICAR (for malonyl-CoA), or 20 mM 2-DG (for NADPH) for 24 h. The concentrations of the malonyl-CoA and NADPH were determined as described in the Materials and Methods section. The graphs represent the results as the fold change versus CTL. **p < 0.01; ***p < 0.05.

Malonyl-CoA is a precursor of fatty acids and its synthesis depends on the ACC activity, which is rapidly inhibited by the phosphorylation of Ser^79^, and metformin induces the phosphorylation of ACC on this residue (Fig. 2, 3). To obtain insights into the molecular events implicated in the rapid action of metformin on lipogenesis, we analyzed the malonyl-CoA concentration. A strong activator of AMPK, 5-aminoimidazole-4-carboxamide ribonucleotide (AICAR), was used as a control to inhibit the ACC activity. The AICAR significantly decreased the malonyl-CoA concentration, whereas the metformin had no effect in the LNCaP and DU145 cell lines (Fig. 5A).

Lipogenesis requires 12 NADPH molecules to synthesize 1 fatty acid molecule. In order to determine whether metformin alters the NADPH levels, we assessed the NADPH content in the prostate cancer cells. An inhibitor of glycolysis, 2-deoxyglucose (2-DG), significantly decreased the NADPH concentration in the LNCaP and DU145 cells. No change in the NADPH was observed in DU145, and the metformin induced a slight but significant decrease in the LNCaP cells (Fig. 5B). Our results indicate that the inhibitory action of metformin is not due to the decrease of malonyl-CoA or NADPH.

### Metformin induces an energy deficiency state in prostate cancer

**Figure 6:**
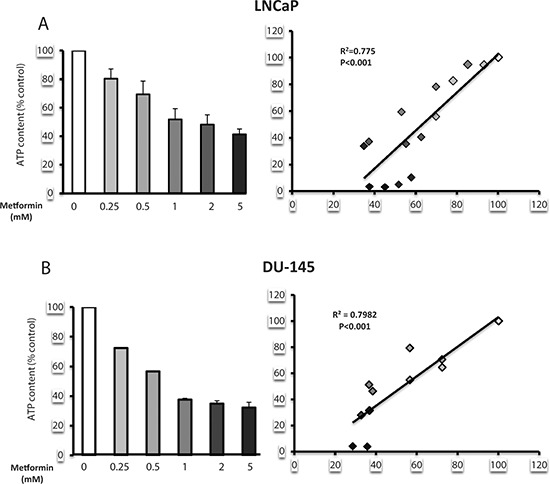
Metformin induces a state of energy deficiency, which correlates with a decrease in lipogenesis **(A, B)** LNCaP and DU145 were treated with increasing concentrations of metformin (0.25 to 5 mM). The ATP content (left panel), expressed as the % of the control, and lipogenesis were monitored. The correlation between the lipogenesis and the ATP content is shown in the left panel. The results are representative of three independent experiments.

Lipogenesis is an energy-demanding process, which consumes 7 molecules of ATP per molecule of palmitate produced [20]. We have shown that metformin inhibits complex 1 of the mitochondrial respiratory chain and decreases the ATP concentration in prostate cancer cells [17]. To evaluate the potential of metformin to affect the energetic state of prostate cancer cells, we assessed the ATP concentration in the LNCaP and DU145 cells after treatment with different concentrations of metformin. The metformin induced a dose-dependent decrease in the ATP content in both cell lines (Fig. 6A). We then asked whether a correlation exists between the inhibition of lipogenesis and the decrease in the ATP concentration. Interestingly, the decrease in the ATP content was significantly correlated with the inhibitory effect of the metformin on lipogenesis, with an *R*^2^ = 0.775 in LNCaP and 0.798 in DU145 (Fig. 6A, 6B).

To highlight the importance of the energetic state of the cell in the control of lipogenesis, we treated the cells with rotenone, which is a strong inhibitor of complex 1 and decreases the ATP content [17]. Similar to metformin, increasing the concentration of rotenone (from 5 to 50 μM) induces a decrease in the ATP concentration, which parallels the inhibition of lipogenesis (Fig. S4). Together, our results suggest that the effect of metformin on lipogenesis in prostate cancer cells is linked to changes in the ATP content rather than regulation of the protein expression.

## DISCUSSION

The anti-diabetic drug metformin has received significant attention lately because of its anti-tumoral action [21]. Depending on the cancer cell type, metformin induces either cell-cycle arrest, apoptosis or autophagy. These cellular effects are associated with major alterations in the cellular metabolism. Metformin acts as a calorie-restriction agent, which forces cells to adapt their metabolism to minimize energy-consuming reactions and optimize energy-producing pathways. For example, metformin, through the inhibition of mTOR, decreases protein synthesis, thus providing a mechanism of action for metformin in the inhibition of cancer-cell proliferation [22]. Similarly, the inhibition of lipogenesis may also be implicated in the anti-proliferative effects of metformin. Indeed, lipogenesis plays a major role in cell proliferation because it generates lipids for the formation of the neo-synthetized membranes of rapidly dividing cells. The increase in lipogenesis is associated with a poor prognosis, tumor growth and aggressiveness, especially in prostate cancer [23].

Targeted therapies using FASN inhibitors, such as C75, decrease the cancer cell proliferation and tumor growth and may offer new therapeutic opportunities for cancer [24, 25]. Androgens drive prostate cancer carcinogenesis and progression, even in androgen-independent tumors where the androgen receptor signaling remains active. Interfering with the androgen receptor pathway is the gold standard for androgen-sensitive tumors and castration-resistant prostate cancer (CPRC). Drugs such as MDV3100 and abiraterone (an inhibitor of androgen synthesis) have provided encouraging results; however, resistance to these agents has recently emerged [26]. Androgen-induced lipogenesis is enhanced during the emergence of androgen independence and contributes to the growth of castration-resistant prostate cancer cells [19].

Lipogenesis is controlled by several proteins, and we demonstrated that metformin decreases the FASN, SREBP1c and ACC expression in LNCaP cells. This is in accordance with previous studies showing that the alteration of SREBP1c affects lipogenesis [27, 28]. Recently, Yecies *et al*. deciphered the molecular mechanism implicated in this regulation and demonstrated that Akt stimulates hepatic SREBP1c through mTOR dependent and independent pathways [29]. AMPK negatively regulates mTOR; it phosphorylates SREBP1c and inhibits its activity to diminish hepatic fatty acid accumulation [30]. Other studies in cancer cells demonstrated that targeting AMPK/mTOR inhibits lipogenesis. MT63–78, a novel selective activator of AMPK, decreases prostate tumor growth and inhibits FASN and SREBP1c expression, and the anti-growth effects of this activator are mediated by the inhibition of lipogenesis [31]. Metformin is an activator of AMPK and an inhibitor of mTOR, and it decreases the expression of FASN and SREBP1c in colonic tumors from mice fed a high-energy diet [32]. Similarly, metformin affected the expression of hepatic FASN and ACC in mice treated with the potent liver carcinogen diethylnitrosamine (DEN). In the same study, the overexpression of a constitutively active form of SREBP1c induced the expression of lipogenic genes and reversed the antiproliferative effects of metformin in several hepatocellular carcinoma cell lines. However, there is no evidence in this study of the restoration of functional lipogenesis. Here, we demonstrated that despite the relatively high level of lipogenic proteins induced by the overexpression of SREBP1c or androgen treatment, the metformin was still able to hamper lipogenesis.

The major source of NADPH in animals is the pentose phosphate pathway, and an increase in glucose consumption stimulates this pathway. Metformin increases glucose uptake in cancer cells [17]; therefore, we were not expecting a decrease in the NADPH concentration. Instead, we showed that NADPH is not altered in DU145, and it is slightly decreased in LNCaP. Therefore, NADPH does not appear to be the limiting factor responsible for the inhibition of lipogenesis, at least in DU145.

Another limiting factor of lipogenesis is the concentration of malonyl-CoA. Acetyl-CoA produces malonyl-CoA, which is converted to palmitic acid by FASN. ACC regulates the synthesis of malonyl-CoA from acetyl-CoA, and its activity is inhibited by AMPK through its phosphorylation at Ser^79^. We demonstrated that metformin does not affect the malonyl-CoA content. Although the activation of AMPK (inhibition of ACC) decreases the synthesis of malonyl-CoA, we can expect that the inhibition of the later stages of lipogenesis induces an accumulation of malonyl-CoA, as demonstrated by Fritz et al. [3]. In addition, in response to metformin, prostate cancer cells were shown to increase their dependence on reductive glutamine metabolism [33]. This pathway leads to the formation of acetyl-CoA, the substrate of ACC, suggesting that the net result of the metformin action is an equilibrium between the accumulation of malonyl-CoA and a partial inhibition of its synthesis.

Metformin inhibits complex 1 of the mitochondrial respiratory chain in intact hepatocytes and cancer cells [17, 34], resulting in a decrease in the ATP concentration and a state of energy deficiency in the cells. In response to this energetic stress, cells increase their glucose consumption and upregulate glycolysis to produce ATP [17, 35]. They also increase their dependence on reductive glutamine metabolism [33]. This pathway provides alpha-ketoglutarate to the TCA cycle to generate succinate, which is oxidized by complex 2, and therefore bypasses complex 1 of the mitochondrial respiratory chain. These compensatory mechanisms are essential to avoid the induction of apoptosis. We demonstrated that the combination of 2-deoxyglucose (2-DG), an inhibitor of glycolysis, and metformin leads to the depletion of ATP and cell death [17]. Similarly, the decrease in the glutamine flux sensitizes cells to the anti-proliferative action of metformin [33].

The exact mechanism involved in the inhibition of complex 1 by metformin is not well understood, but it is well established that the inhibition of gluconeogenesis in hepatocytes results from a disruption of the energy metabolism [36, 37]. This result is supported by the correlation observed between the reduction of the ATP/ADP level in the inhibition of gluconeogenesis in hepatocytes [36]. It is worth noting that the ATP level was significantly reduced in primary hepatocytes treated with 0.25 mM of metformin, and a higher concentration induced a stronger decrease. In accordance with these results, we showed that metformin induces a decrease in the ATP level in a dose-dependent manner, which significantly correlates with the inhibition of lipogenesis.

Our work highlights a new function for metformin in its anti-tumoral action. In addition, as a calorie-restricting agent, it unravels a direct relationship between the energy status of the cells and the fatty acid synthesis.

## MATERIALS AND METHODS

### Cell lines and culture conditions

The cell lines were purchased from the ATCC (Manassas, VA, USA). The LNCaP cells were cultured in RPMI 1640 medium, and the PC-3 and DU145 cells were cultured in DMEM (Invitrogen, Carlsbad, CA, USA) containing 25 mmol/L glucose supplemented with 10% fetal bovine serum (FBS) and 100 units/mL penicillin at 37°C and 5% CO_2_.

### Chemicals

The metformin and 2-deoxyglucose (Sigma Chemical Co., St. Louis, MO, USA) were dissolved in the culture media. Compound C and rotenone were purchased from Calbiochem (Merck, Darmstadt, Germany). R1881 (Sigma Chemical Co., St. Louis, MO, USA) was dissolved in DMSO.

### Cell infection

The cells were infected with an empty adenoviral vector or the adenoviral construct of a transcriptionally active form of SREBP-1c (a kind gift from Dr. F. Foufelle, INSERM U872, Paris) for 24 h. The medium was replaced and the cells were then treated and used for western analysis, lipogenesis and proliferation experiments.

### Western blotting

The cell extracts were prepared using lysis buffer [15], and the immunoblotting was performed using antibodies against ACC (Cell Signaling Technology); Ser^79^ P-ACC (Millipore), FASN and HSP90 (Santa Cruz Biotechnology); SREBP1c (BD Biosciences); and α-tubulin (Sigma Chemical Co.).

### Quantification of the lipogenesis

Subconfluent cell cultures were grown in 6-well plates and treated with 5 mM metformin for 24 h. The cultures were then incubated with [^3^H] acetate (0.2 μCi/ml) for 1 h. The incorporation of [^3^H] acetate into the lipids was measured at the end of the incubation after the addition of toluene-based scintillation fluid [16]. The counts per minute (cpm) were normalized to the protein content in the total cell lysate.

### Malonyl-CoA quantification

The quantification of the malonyl-CoA concentration in the whole-cell lysates from the LNCaP and DU145 cells was performed via an enzyme-linked immunosorbent assay using a commercial kit according to the manufacturer's instructions (Antibodies online.com). Briefly, subconfluent cell cultures grown in 6-well plates were treated, and the whole-cell lysates were subjected to several freeze-thaw cycles and then centrifuged. The malonyl-CoA in the supernatant was detected using a specific biotin-conjugated antibody on pre-coated 96-well plates and was revealed with avidin-conjugated horseradish peroxidase. The values are expressed in nanomoles of malonyl-CoA and are normalized to the total protein content.

### NADPH quantification

The cells were treated with 5 mM metformin or 20 mM 2-deoxyglucose for 24 h. The quantification of the NADPH concentration in the whole-cell lysates from the LNCaP and DU145 cells was performed using a commercial kit according to the manufacturer's instructions (AAT Bioquest, Sunnyvale, CA, USA).

### Measurement of the ATP concentration

The quantification of the ATP concentration from the LNCaP and DU145 cells treated with different concentrations of metformin or rotenone was performed using a commercial kit according to the manufacturer's instructions (Roche, Bale, Switzerland). The ATP concentration was normalized to the protein content.

### Quantitative real-time PCR

For the gene expression analysis following the treatment of the cells with metformin for 24 h, an RNA isolation, reverse transcription (RT) and quantitative (q) real-time polymerase chain reaction (PCR) were carried out as described previously [10]. The qPCR was conducted using a Step-one Real Time PCR system (Applied Biosystems, Life Technologies SAS, Villebon sur Yvette, France) with SYBR Green Master Mix (Applied Biosystems, Life Technologies SAS) and oligonucleotides specific for human ACC1, ACC2, SREBP1c and FASN. The values are expressed as the relative mRNA level of the specific target gene normalized to the HPRT levels, which was obtained using the formula 2^−(ΔΔCt)^.

## SUPPLEMENTAL MATERIAL AND METHODS


