# Supplementary material for: Metformin-induced energy deficiency leads to the inhibition of lipogenesis in prostate cancer cells
Source: Oncotarget. 2015 Mar 10;6(17):15652–61. doi: 10.18632/oncotarget.3404 (PMC4558177; doi:10.18632/oncotarget.3404)
Supplement: Supplementary file 1 [file oncotarget-06-15652-s001.pdf]

SUPPLEMENTAL MATERIAL AND METHODS

Luciferase assay

LNCaP were transfected using lipofectamin reagent (Invitrogen, Life technology) with 1 µg of each of plasmid, according to the manufacturer's instructions. Forty-eight hours after transfection, in presence or absence of 5 mM metformin, cells were lysed and luciferase activity was assessed.

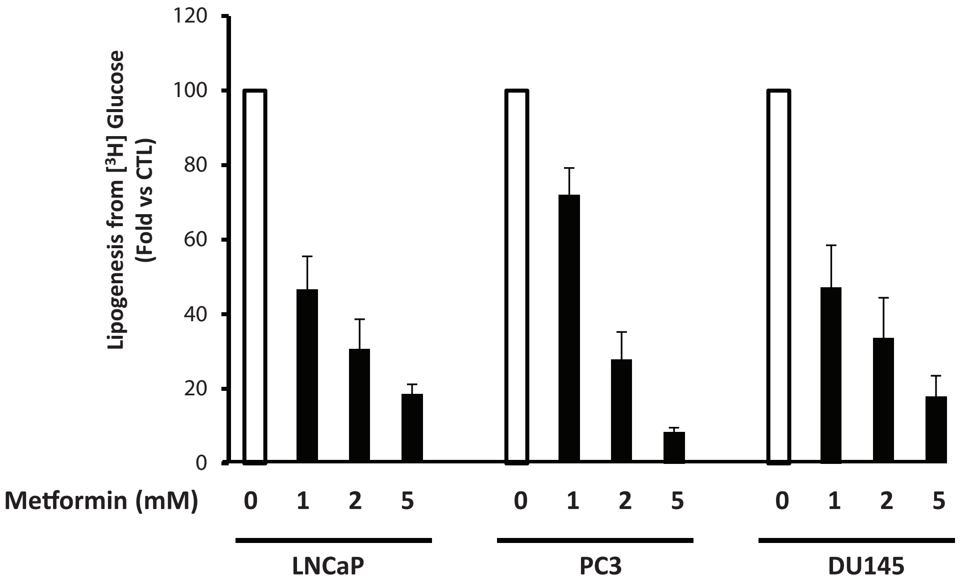

Supplementary Figure S1: Metformin inhibits lipogenesis from ^{3}H Glucose. Cells were treated with different concentrations of metformin (1 to 5 mM) for 24 h and the lipogenesis was performed as described in material and methods using 0.2 μCi/ml of [^{3}H] Glucose.

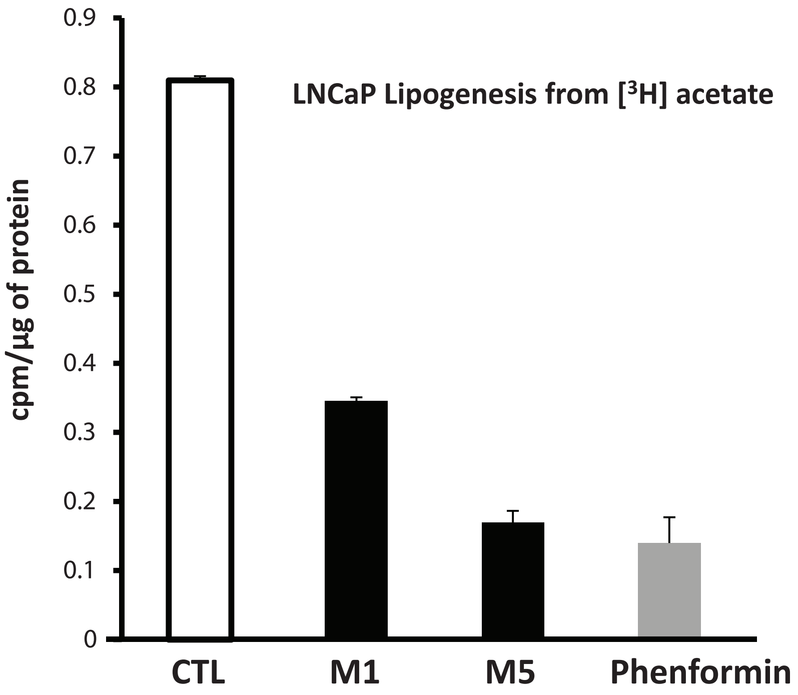

Supplementary Figure S2: Phenformin inhibits lipogenesis. The cells were treated with different concentrations of metformin 1 mM: M1, or 5 mM: M5 or 100 µM phenformin for 24 h and lipogenesis from [^{3}H] Acetate was performed as described in Material and Methods.

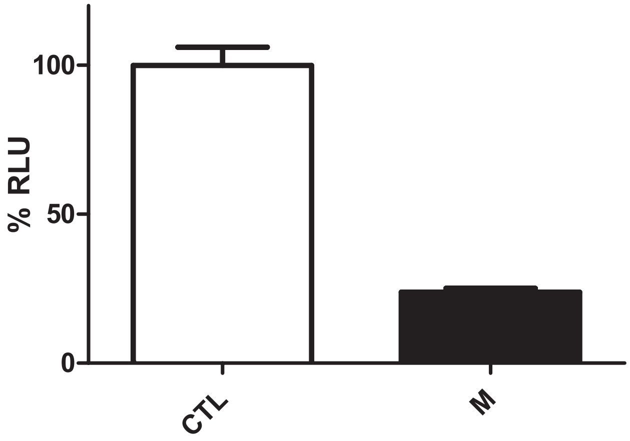

Supplementary Figure S3: Metformin inhibits SREBP1c transcriptional activity. LNCaP cells were transfected with a FASN promoter-Luc construct (−135pFAS-Luc) , a kind gift of F. Foufelle. 24 h after the transfection cells were treated for 3 h with 5 mM metformin (M) and luciferase activity was measured.

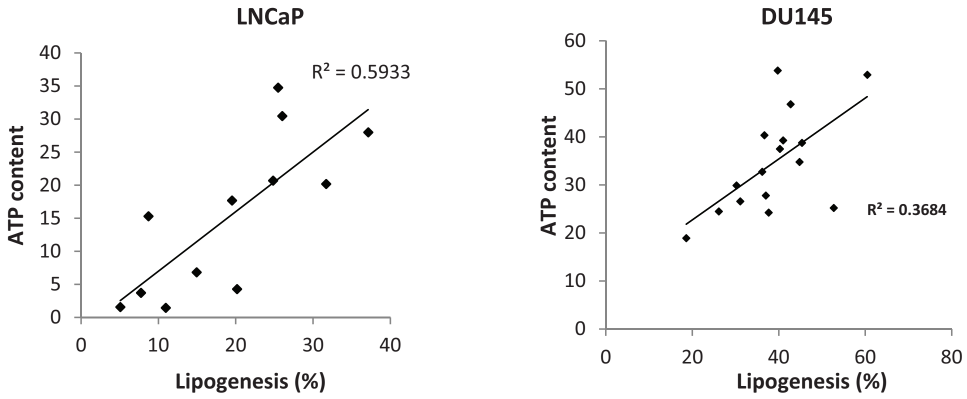

Supplementary Figure S4: Rotenone decreases ATP content which correlates with a decrease of lipogenesis. **(A, B)** LNCaP and DU145 were treated with increasing concentrations of rotenone (5, 10, 20 or 50 µM), the graph represents the correlation between lipogenesis and ATP content.
